# Quality assurance and characterization of narrowband ultraviolet B devices for use at home: lessons from the HI‐Light Vitiligo Trial

**DOI:** 10.1111/bjd.19630

**Published:** 2020-11-29

**Authors:** A. Rogers, P. Akram, J.M. Batchelor, J. Crutchley, M. Grocki, R.H. Haines, G. Meakin, K. O’Dowd, J. Ravenscroft, K.S. Thomas

**Affiliations:** ^1^ Department of Medical Physics and Clinical Engineering Nottingham University Hospitals NHS Trust Nottingham NG7 2UH UK; ^2^ Centre of Evidence Based Dermatology King’s Meadow Campus Lenton Lane Nottingham NG7 2NR UK; ^3^ Nottingham Clinical Trials Unit University of Nottingham Queen’s Medical Centre Nottingham NG7 2UH UK; ^4^ Department of Dermatology Nottingham University Hospitals NHS Trust Nottingham NG7 2UH UK

dear editor, Handheld narrowband ultraviolet B (NB‐UVB) devices are available for the treatment of various skin conditions, but their optical radiation omissions are often not thoroughly characterized. Following a pilot study^1^ that identified potential dosimetry issues, we separately characterized and quality controlled all devices issued to participants in the main Home Interventions and Light therapy for the treatment of Vitiligo Trial (HI‐Light Vitiligo Trial; ISRCTN 17160087).^2^, ^3^, ^4^, ^5^

The handheld NB‐UVB device used in the HI‐Light Vitiligo Trial was the Dermfix 1000MX unit using a LightTech LTC 9W/G23 tube (Androv Medical, Leatherhead, UK). For the spectral emission measurements, we used a Bentham DMc150 spectroradiometer (Bentham Instruments, Reading, UK) and for the irradiance measurements an ILT1700 radiometer and SED005 UVB detector (International Light Technologies, Peabody, MA, USA). The spectroradiometer was calibrated against a mercury lamp with National Physical Laboratory traceable spectral emissions for both wavelength and spectral irradiance. The ILT1700 radiometer and detector were field calibrated monthly against the spectroradiometer during the study.

The characterization identified the device irradiance measured at 3 min including a 1‐min warm‐up, spectral emissions, and the variance of irradiance across an initial sample of 10 devices. The spectral emissions of the devices showed good agreement with the manufacturer’s specification, with a main emission at 313 nm and subsidiary peaks at 365 nm, 405 nm and 435 nm. The device irradiance was measured for 10 devices at the comb tip in the middle of the tube. The mean (SD) of the 10 measurements was found to be 3·8 (0·22) mW cm^−2^, compared with the manufacturer’s specification of 7 mW cm^−2^. This significant deviation from specification required an adjustment to the HI‐Light trial treatment schedule.^6^ We also considered the variation in output across the sample of 10 devices. All fell within our clinically determined rejection criterion of ± 20%. Simulation of a 9‐month course of treatment for three vitiligo patches in an individual with skin type VI was carried out with a number (*n*) of devices, and the mean drop in output was 23% (SD 8·8) after one‐third of the treatment course (*n* = 6), 29% (SD 6·7) after two‐thirds (*n* = 4) and 34% (SD 5·3) after the full treatment course (*n* = 2).

During the trial, quality control checks of all devices prior to issue to participants determined whether the spectral emissions remained the same as at characterization, whether device output was within our predetermined range and whether each device was electrically safe. Devices were tested in batches of 15–25. In total 54 of 425 active devices (13%) were rejected as having an output outside the criterion of 4 mW cm^−2^ ± 20%.

The characterization results show the importance of testing devices prior to determining the treatment schedule in the trial. The 43% discrepancy between output specification (7 mW cm^−2^) and measured output (4 mW cm^−2^) required adjustment of the treatment schedule. The drop in output of a device over time was as expected (Figure 1). In clinical practice this would simply mean that over time the user would need to move to a higher step of the treatment schedule in order to achieve a therapeutic dose.

**Figure 1:**
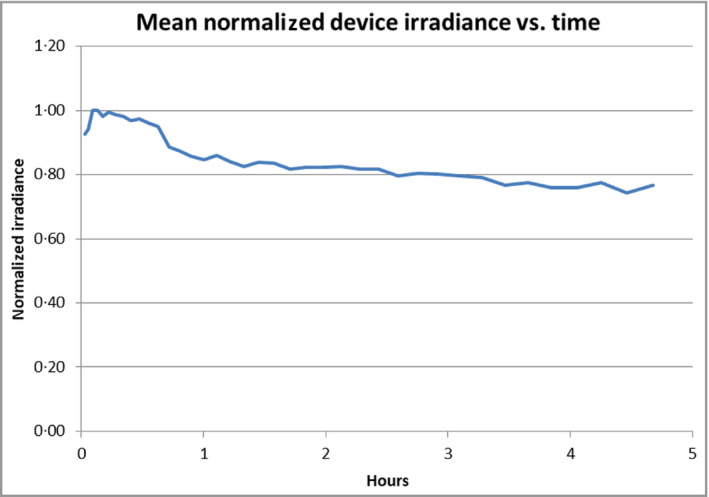
Mean normalized irradiance (normalized to maximum irradiance) vs. time (hours) for six devices. The SD was approximately ± 12% of the normalized value at the start, ± 15% at 2 h and ± 18% at 4 h.

The fact that one in eight devices was rejected due to their output lying outside the ± 20% cutoff shows the importance of measuring device output. The minimum device irradiance was 2·4 mW cm^−2^ and the maximum was 5·0 mW cm^−2^. This quality control reduced the variance in treatment exposure attributable to device output and demonstrates the usefulness of pretreatment checks. Furthermore, given that these devices may be purchased by members of the public, the output variation from specification and its variation between tubes shows the need for clinical and technical supervision, backed up by robust quality assurance processes, during their use. No devices were rejected due to spectral emissions or electrical safety.

The device tests described in this paper require expensive UV test equipment and scientific and technical expertise to interpret the results. These staff and equipment are not available at all hospitals, so it may be necessary to develop specialist centres that provide support to several dermatology services in a regional approach.

Our findings regarding the dosimetry and performance of handheld NB‐UVB units will help to inform the design of community‐based phototherapy services in the future. However, there are additional considerations regarding such services.

## Author Contribution

**Andy Rogers:** Conceptualization (lead); Data curation (equal); Formal analysis (equal); Funding acquisition (supporting); Investigation (equal); Methodology (equal); Writing‐original draft (lead); Writing‐review & editing (lead). **Akram Perways:** Conceptualization (equal); Data curation (equal); Formal analysis (equal); Funding acquisition (supporting); Investigation (equal); Methodology (equal); Project administration (equal); Writing‐original draft (supporting); Writing‐review & editing (supporting). **Jonathan Batchelor:** Conceptualization (equal); Data curation (equal); Formal analysis (equal); Funding acquisition (lead); Investigation (equal); Methodology (equal); Writing‐original draft (equal); Writing‐review & editing (equal). **Janine Crutchley:** Conceptualization (equal); Data curation (equal); Formal analysis (equal); Investigation (equal); Methodology (equal); Writing‐original draft (equal); Writing‐review & editing (equal). **Mariusz Grocki:** Conceptualization (equal); Data curation (equal); Formal analysis (equal); Investigation (equal); Methodology (equal); Writing‐original draft (equal); Writing‐review & editing (equal). **Rachel Haines:** Conceptualization (equal); Data curation (equal); Formal analysis (supporting); Funding acquisition (equal); Investigation (equal); Methodology (equal); Project administration (equal); Writing‐original draft (supporting); Writing‐review & editing (equal). **Garry Meakin:** Conceptualization (equal); Data curation (equal); Formal analysis (supporting); Funding acquisition (equal); Investigation (equal); Methodology (equal); Project administration (equal); Writing‐original draft (supporting); Writing‐review & editing (supporting). **Kenneth O'Dowd:** Conceptualization (equal); Data curation (equal); Formal analysis (equal); Investigation (equal); Methodology (equal); Writing‐original draft (equal); Writing‐review & editing (equal). **Jane Ravenscroft:** Conceptualization (equal); Data curation (equal); Formal analysis (equal); Investigation (equal); Methodology (equal); Writing‐original draft (equal); Writing‐review & editing (equal). **Kim S Thomas:** Conceptualization (equal); Data curation (equal); Formal analysis (equal); Funding acquisition (lead); Investigation (equal); Methodology (equal); Writing‐original draft (equal); Writing‐review & editing (equal).
